# CDC42EP3 promotes colorectal cancer through regulating cell proliferation, cell apoptosis and cell migration

**DOI:** 10.1186/s12935-021-01845-8

**Published:** 2021-03-16

**Authors:** Qiang Feng, Dongkui Xu, Mingyao Zhou, Zijian Wu, Zhiyuan Wu, Zheng Wang, Jianjun Bi, Wei Pei

**Affiliations:** 1 Department of Colorectal Surgery, National Cancer Center/National Clinical Research Center for Cancer/Cancer Hospital, Chinese Academy of Medical Sciences and Peking Union Medical College, Beijing, 100021 China; 2 Department of VIP, National Cancer Center/National Clinical Research Center for Cancer/Cancer Hospital, Chinese Academy of Medical Sciences and Peking Union Medical College, Beijing, China; 3 Department of Immunology, National Cancer Center/National Clinical Research Center for Cancer/Cancer Hospital, Chinese Academy of Medical Sciences and Peking Union Medical College, Beijing, China; 4 State Key Laboratory of Molecular Oncology, National Cancer Center/National Clinical Research Center for Cancer/Cancer Hospital, Chinese Academy of Medical Sciences and Peking Union Medical College, Beijing, China

**Keywords:** Colorectal cancer, CDC42EP3, EMT, Cell proliferation, Cell apoptosis

## Abstract

**Background:**

Nowadays, colorectal cancer (CRC) is one of the most commonly diagnosed malignant tumors worldwide, the incidence rate of which is still increasing year by year. Herein, the objective of this study is to investigate whether CDC42EP3 has regulatory effects in CRC.

**Methods:**

First, CDC42EP3 knockdown cell model based on HCT116 and RKO cell lines was successfully constructed, which was further used for constructing mouse xenotransplantation models. Importantly, effects of CDC42EP3 knockdown on proliferation, colony formation, apoptosis, and migration of CRC were accessed by MTT assay, EdU staining assay, colony formation assay, Flow cytometry, and Transwell assay.

**Results:**

As the results, we showed that CDC42EP3 was significantly upregulated in CRC, and its high expression was associated with tumor progression. Furthermore, knockdown of CDC42EP3 could inhibit proliferation, colony formation and migration, and promote apoptosis of CRC cells in vitro. In vivo results further confirmed knockdown of CDC42EP3 attenuated tumor growth in CRC. Interestingly, the regulation of CRC by CDC42EP3 involved not only the change of a variety of apoptosis-related proteins, but also the regulation of downstream signaling pathway.

**Conclusion:**

In conclusion, the role of CDC42EP3 in CRC was clarified and showed its potential as a target of innovative therapeutic approaches for CRC.

## Background

Colorectal cancer (CRC) is a prevalent cancer type with a high incidence and mortality rate in the colon or rectum, and is the fourth leading cause of cancer-related death worldwide [1, 2]. According to statistics, approximately a quarter of CRC patients are diagnosed at an advanced disease stage, leading to miss the appropriate time for surgical resection [3, 4]. In the past decades, the exploration of molecular mechanism and the screening of therapeutic targets have become the focus of colorectal cancer research [5, 6]. Nowadays, although the combination of targeted therapy and chemotherapy has made the management and treatment of metastatic colorectal cancer significantly improved to a certain extent, the 5-year survival rate of only 10–20% and the patient’s quality of life are still unsatisfactory [7]. Therefore, exploring more promising targets and their mechanistic roles in CRC not only could deepen the understanding of the pathogenesis of CRC, but contribute to the clinical diagnosis and treatment of CRC as well.

Cdc42 (cell division cycle 42) a member of Rho GTPase protein family, is a highly conserved protein. Cdc42 plays an important role in a variety of cellular activities, such as cell polarization, cytoskeleton remodeling, cell transformation, regulation of filiform pseudopodia formation, neural development and so on [8]. Moreover, recent studies have further revealed that it is closely related to proliferation, migration and invasion of tumor cells, thus regulating the aggressive growth and metastasis of malignant tumors [9]. For example, Yang et al*.* reported that Cdc42 exhibited relatively high expression in pancreatic cancer, could facilitate the fast growth of tumor, and showed significant correlation with prognosis of patients [10]. Cdc42 was also described as a key mediator in the regulation of several types of human cancers, such as gastric cancer and cervical cancer, induced by microRNAs [11, 12]. CDC42EP3 is a member of Cdc42 effector protein family, whose biological functions in human diseases were rarely reported. Some studies showed that CDC42EP3 plays a role in the function of cancer-associated fibroblasts and DNA damage repair, both of which could be adjusted Cdc42 [13–15]. Noteworthy, although the overexpression of Cdc42 in colorectal cancer and its clinical relevance have been studied to some extent [16], the role of CDC42EP3 in colorectal cancer has not been reported, which attracted our attention.

As far as we know, this study is the first one to comprehensively investigate the role of CDC42EP3 in colorectal cancer. The silencing of CDC42EP3 in colorectal cancer cell lines further clarified its effects on the phenotype of colorectal cancer cells such as cell proliferation, apoptosis, cell cycle and cell migration. Thereafter, the in vivo experiments where mice xenograft model was constructed were performed for subsequent verification. Herein, this study demonstrated that CDC42EP3 may play a role as a tumor promotor in the development and progression of colorectal cancer, which provided a reference for CDC42EP3 as a novel therapeutic target in the treatment of colorectal cancer.

## Methods

### Clinical tissues and cell culture

CRC tissues and para-normal tissues microarray chip was obtained from Shanghai Outdo Biotech Company (#HColA180Su15) including 97 cancer tissues and 75 normal tissues collected from CRC patients during January 2007.1 to August 2015.7. The approval and written informed consent were obtained from all patients involved.

Human colorectal cancer cell line HCT116, RKO, SW480 and Caco2 were purchased from BeNa Technology (Hangzhou). HCT116, RKO and SW480 cells were maintained in DMEM medium supplemented with 10% FBS and Caco2 cells were grown in RPMI-1640 medium supplemented with 10% FBS. All cells were humidly cultured in a 37 °C 5% CO_2_ incubator with culture medium changed every three days.

### shRNA design and lentivirus construction

Three target sequences of CDC42EP3 were designed as follow: 5′- CGGACTCTGTGTTCACAGAAA-3′, 5′-AAGCTCTCATGTTGCCCTTAT-3′, 5′- ATGCGAGCTCATCAAGGGAAA-3′. shRNAs targeting CDC42EP3 silence based on the above sequences were further designed and prepared by Shanghai Bioscienceres Co., Ltd. (Shanghai, China). Then the shRNA sequences were cloned into BR-V-108 linearized vector using Fermentas T4 DNA Ligase (Thermo Fisher Scientific). Verified lentiviruses were transfected into Top 10 E.coli receptor cells for amplifying and high purity plasmid (EndoFree midi Plasmid Kit, TIANGEN) were extracted. 293 T cells were used for packaging and lentiviral harvest was performed 72 h after transfection.

### Immunohistochemistry (IHC) analysis of CDC42EP3

Slides containing CRC tissues and normal tissues were used for immunohistochemistry. In brief, slides were baked at 60 °C for 1 h. After cooling, slides were dehydrated in xylene and rehydrated in graded alcohol and the activity of endogenous antigen were revealed in boiling citric acid buffer. Then slides were blocked with 3% H_2_O_2_ and rabbit serum successively. Anti-CDC42EP3 primary antibody (1:50, # ab122869) were added for incubating at 4 °C overnight. After washed with PBS, HRP Goat anti rabbit IgG (1:400, # ab6721, Abcam) was added and incubated for 2 h at room temperature. DAB solution and hematoxylin was used for coloring. All slides were pictured at 200 × and 400 × objective and then were viewed with ImageScope and CaseViewer. IHC scores were determined by staining percentage scores and staining intensity scores. Staining percentage scores were classified as: 1 (1%-24%), 2 (25%-49%), 3 (50%-74%), 4 (75%-100%) and staining intensity was scored as 0 (signalless color), 1 (brown), 2 (light yellow), 3 (dark brown). High/low CDC42EP3 expression was defined based on the median of IHC score of all tissue samples.

### qRT-PCR

Total RNA from shCDC42EP3 and shCtrl RKO and HCT116 cells were extracted according to sigma Trizol instructions (Invitrogen). cDNA was obtained by RNA reverse transcription and the qRT-PCR was performed (AceQ qPCR SYBR Green Master Mix) subsequently, according to the instructions of Hiscript QRT supermix for qPCR (Vazyme). GAPDH was used as a reference control. Primer sequences were as follows:

CDC42EP3 upstream primer, 5′-AGCAGTCTGTTGGAGAATGGG -3′, CDC42EP3 downstream primer, 5′-AGGAGGGAACCTGTAAGGTCAG-3′, GAPDH Primer, 5′-TGACTTCAACAGCGACACCCA-3′, GAPDH Primer, 5′-CACCCTGTTGCTGTAGCCAAA-3’.

### Western Blotting

Total protein from shCDC42EP3 and shCtrl RKO and HCT116 cells were extracted by ice-cold RIPA buffer (Millipore) and the concentration was detected by BCA Protein Assay Kit (HyClone-Pierce). Next, 20 μg total protein was separated by 10% SDS-PAGE (Invitrogen) and transferred onto PVDF membranes. The membranes were blocked with TBST solution containing 5% non-fat milk at room temperature for 1 h and then were incubated at 4 °C overnight with anti-CDC42EP3, anti-N-cadherin, anti-Vimentin, anti-Snail, anti-E-cadherin, anti-GAPDH (Abcam). After washing, the membrane was further incubated with second antibody Goat anti-rabbit IgG (1:3000, Beyotime) for 2 h at room temperature. The proteins bands were visualized by enhanced chemiluminescence (ECL, Amersham) and the density was analyzed.

### MTT assay

2000 per well RKO and HCT116 cells in shCDC42EP3 and shCtrl groups were seeded into a 96-well plate with 100 mL culture medium. Four hours before detection at 1, 2, 3, 4, and 5 days after seeding, 20 μL 5 mg/mL MTT solution (GenView) was added for coloring. After formazan was dissolved by DMSO solution, the optical density (OD) was measured at 490 nm with a reference wavelength of 570 nm.

### Cell apoptosis

shCDC42EP3 or shCtrl infected RKO and HCT116 cells were seeded in a 6-well plate until cell density reached 85% and cultured for 5 days. Cells were harvested and washed with 4 °C ice-cold PBS. After cells were resuspended at a density of 6 × 10^5^ and stained by Annexin V-APC (eBioscience) at room temperature without light for 15 min, binding buffer was added for measure using FACSCalibur (BD Biosciences).

### Colony formation assay

Logarithmic growth phased shCDC42EP3 and shCtrl RKO and HCT116 cells in were seeded into a 6-well plate (1000 cells/well). After photographed by fluorescence microscopy, the cell clones were fixed by 4% paraformaldehyde and stained with 500 μL Giemsa (DingGuo Biotechnology). Clones was observed and numbers of colony was counted (a cluster including more than 50 cells as a colony) under microscope.

### Transwell assay

shCDC42EP3 and shCtrl groups of RKO and HCT116 cells were cultured in a 24-well plate (5 × 10^4^ cells/well) with 100 μL serum-free medium in each well and put the plate in the upper chamber. 600 μL medium supplemented with 10% FBS was added into the lower chamber. Cells were incubated for 24 h at 37 °C with 5% CO_2_. Next, the chamber was upside down on the blotting paper to remove the medium and gently remove the non-metastatic cells. Finally, lower chamber cells were fixed by 4% formaldehyde and stained by Giemsa and solved by 10% acetic acid. Cells from five random fields were selected for observing and the migration ability of cells was analyzed.

### Human apoptosis antibody array

To value the molecular changes involved in CDC42EP3 mediated apoptosis, shCtrl or shCDC42EP3 HCT116 cells were solubilized in ice-cold RIPA buffer (Millipore) and protein concentration was detected by BCA Protein Assay Kit (HyClone-Pierce). Then the samples were subjected to Abcam’s Human Apoptosis Antibody Array-Membrane (# ab134001) according the manufacturer’s instruction. Membrane intensity was acquired using enhanced ECL (Amersham) and signal densities were measured using ImageJ software (National Institute of Health).

### Xenograft animal model experiments

10 mice (4-week-old, female BALB/c nude mice, weighted about 20 g) in two experimental group were subcutaneously injected with shCDC42EP3 and shCtrl RKO cells (4 × 10^6^), respectively, for in vivo tumorigenicity. All mice were obtained from Shanghai Lingchang Laboratory Animal Technology Co., Ltd and housed on a 12-h dark/light cycle at 25 °C with a humidity of 60% conditioned room, with free access to food and water. The tumor size was recorded as L and W (L: longest dimension, W: dimension perpendicular to length, volume of tumor = π/6 × L × W^2^). Before all mice were sacrificed, the anesthetized mice were put under the Berthold Technologies living imaging system for in vivo bioluminescence imaging. Then mice were sacrificed by cervical dislocation and the tumor tissues were harvested. The animal experiments were approved by Institutional Animal Care and Use Committee of National Cancer Center/Cancer Hospital, PUMC&CAMS.

### Ki-67 staining assay

After soaked in 10% formalin for 24 h, fresh mice tumor tissues were paraffin-embedded. Slides were cut and immersed in xylene and ethanol respectively for deparaffinization and rehydration. Sections were incubated with primary antibody Ki-67 (1:200, # Ab16667, Abcam) at 4 °C overnight. Then were incubated with HRP goat anti-rabbit IgG (1:400, # Ab6721, Abcam) at room temperature for 2 h. Slides were stained by Hematoxylin and Eosin (Baso). Stained slides were observed at 100 × and 200 × objective lens microscopic.

### Statistical analyses

Data were shown as mean ± SD. The significance of the differences between shCDC42EP3 and shCtrl group in each experiment was determined using the two-tailed Student’s t test using GraphPad Prism 6.01 (Graphpad Software) and with P value < 0.05 as statistically significant. Mann–Whitney U analysis and Spearman rank correlation analysis were used while explaining the relationships between CDC42EP3 expression and tumor characteristics in patients. All cell experiments were performed in triplicate.

## Results

### CDC42EP3 is upregulated in Colorectal Cancer

**Fig. 1:**
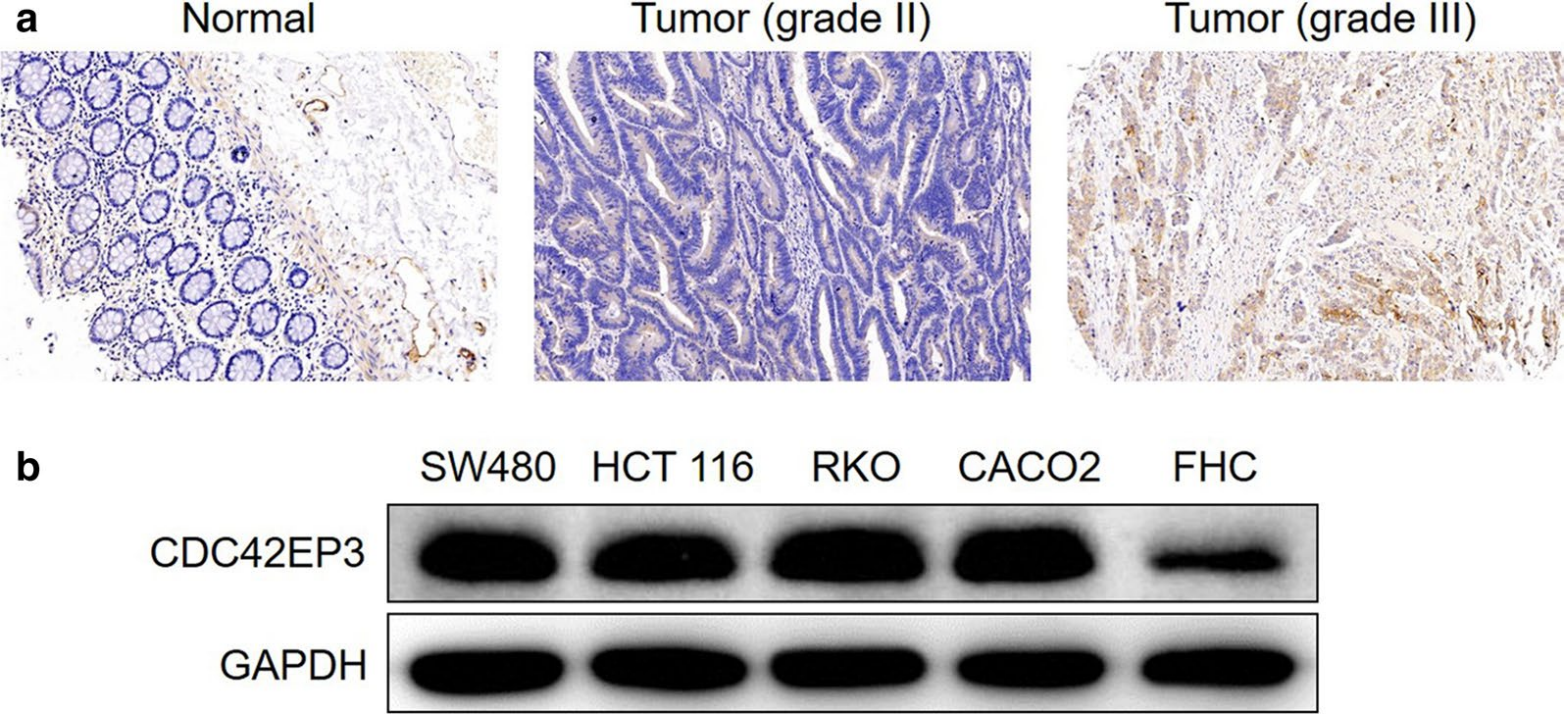
CDC42EP3 was highly expressed in colorectal cancer. **a** The expression of CDC42EP3 in colorectal cancer tissues was examined by IHC analysis and compared with normal skin tissues. **b** The background expression of CDC42EP3 in colorectal cancer cell lines and normal human colorectal mucosal cell line FHC was detected by western blotting

In order to better understand the relationship of CDC42EP3 in the development of colorectal cancer, IHC analysis was undertaken on 97 colorectal cancer tissues and 75 normal tissues as a means to identify observable CDC42EP3 expressions. CDC42EP3 was identified in both normal tissues and colorectal cancer tissues, showing obviously higher expression in colorectal cancer tissues (see Fig. 1a and Table 1). Tumor tissues with advanced grades were also accompanied with upregulated expression of CDC42EP3, demonstrating a potential linkage between the two (see Fig. 1a). CDC42EP3 expression and the connection to tumor characteristics of colorectal cancer patients displayed a correspondence between the appearance of CDC42EP3 and tumor grade in addition to more mesenteric lymph nodes (Table 2). These findings were verified by undertaking Spearman Rank correlation analysis (*P* < 0.05, Table 3). Additionally, western blotting was used to detect the background appearance of CDC42EP3 in colorectal cancer cell lines and normal human colorectal mucosal cell line FHC. As shown in Fig. 1b, the expression of CDC42EP3 was distinctly higher in colorectal cancer cell lines than normal cell line. CDC42EP3′s upregulation emphasizes its possible role in the growth of colorectal cancer.

**Table 1:** Expression patterns of CDC42EP3 in colorectal cancer tissues and normal tissues revealed in immunohistochemistry analysis

CDC42EP3 expression	Tumor tissue		Normal tissue	
	Cases	Percentage	Cases	Percentage
Low	63	64.9%	75	100%
High	34	35.1%	0	/

*P* < 0.001

**Table 2:** Relationship between CDC42EP3 expression and tumor characteristics in patients with colorectal cancer

Features	No. of patients	CDC42EP3 expression		*P* value
		Low	high	
All patients	97	63	34	
Age (years)				0.200
≤ 71	47	27	20	
> 71	44	31	13	
Gender				0.922
Male	53	34	19	
Female	43	28	15	
N stage				0.379
N0	57	39	18	
N1	27	17	10	
N2	11	6	5	
Number of mesenteric lymph nodes				0.022
< 7	42	32	10	
≥ 7	44	23	21	
Grade				< 0.001
II	50	47	3	
III	47	16	31	
T Stage				0.796
T1	1	1	0	
T2	5	4	1	
T3	74	47	27	
T4	13	9	4	
AJCC stage				0.313
1	5	4	1	
2	52	35	17	
3	36	21	15	
4	3	2	1	
Tumor size				0.671
< 5.5 cm	48	32	16	
≥ 5.5 cm	48	30	18

**Table 3:** Relationship between CDC42EP3 expression and tumor characteristics in patients with colorectal cancer analyzed by Spearman rank correlation analysis

Tumor characteristics	index	
Number of mesenteric lymph nodes	Spearman correlation	0.249
	Significance (two tailed)	0.021
	n	86
Grade	Pearson correlation	0.628
	Significance (two tailed)	< 0.001
	n	97

### Building of CDC42EP3 knockdown colorectal cancer cell lines

**Fig. 2:**
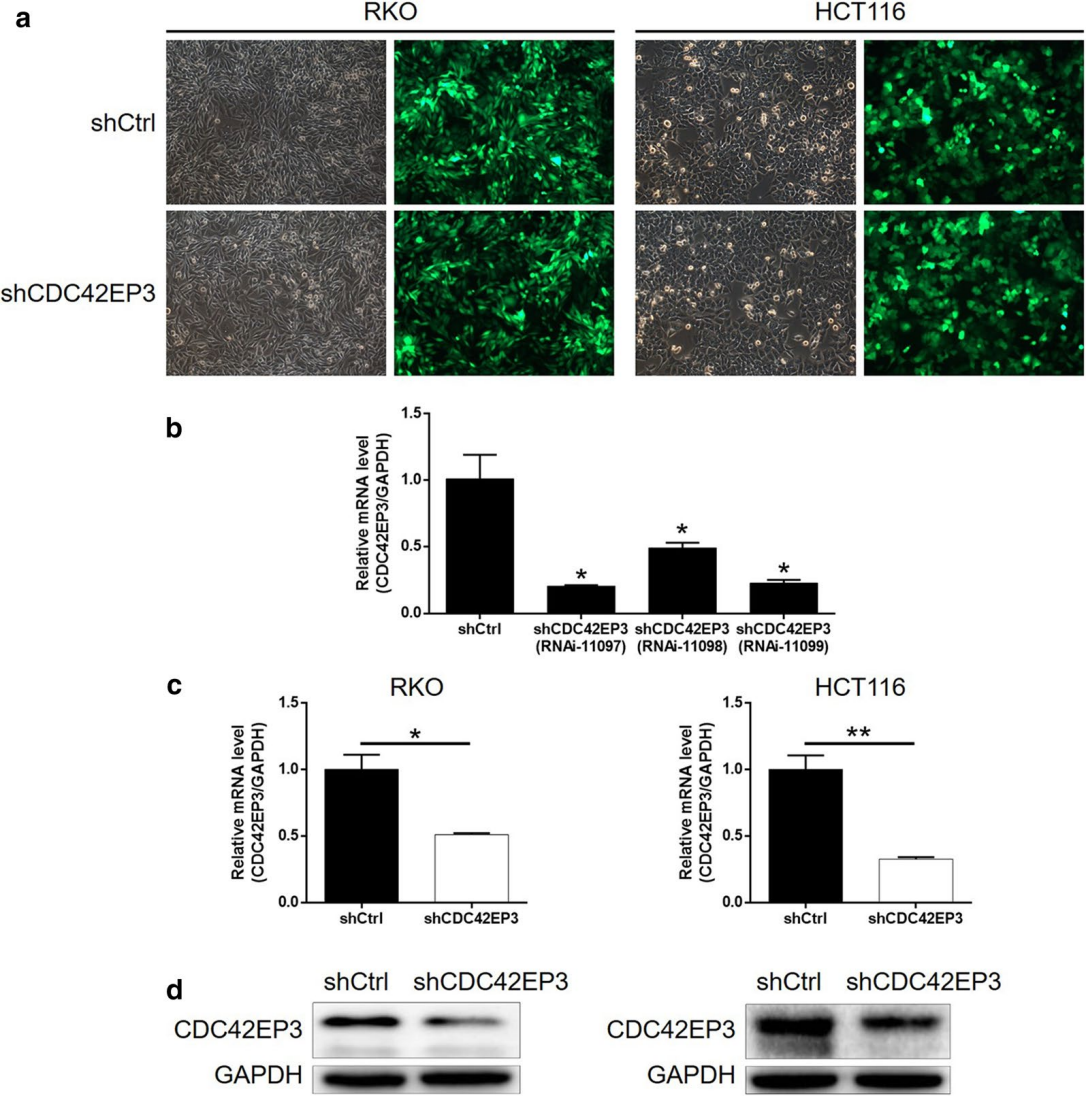
Construction of colorectal cancer cell models with CDC42EP3 knockdown. **a** The fluorescence inside cells was observed and used to represent the transfection efficiency of shCDC42EP3 and shCtrl. **b** The efficiency of 3 shRNAs targeting CDC42EP3 was evaluated by qCPR. The knockdown efficiency of CDC42EP3 in RKO and HCT116 cells was evaluated by qPCR (**c**) and further verified by western blotting (**d**). The representative images were randomly selected from at least 3 independent experiments in duplicate. *P < 0.05, **P < 0.01, ***P < 0.001

As part of our experimentation, we silenced CDC42EP3 in RKO and HCT116 cells in order to better understand its role in development of colorectal cancer in vitro. 3 shRNAs targeting CDC42EP3 (shCDC42EP3), in combination with shCtrl as a negative control, was integrated into lentivirus vector and used for cell transfection. Transfection efficiency, represented using a green fluorescent protein on a lentivirus vector, indicated results of > 80%, in addition to successful transfection (Fig. 2a). Figure 2b reflects that RNAi-11097 was screened as the most effective candidate shRNA for silencing CDC42EP3. This approach was utilized in subsequent experiments. The qPCR results showed that the mRNA level of CDC42EP3 was lowered by 60% and 90% in RKO and HCT116 cells, respectively (*P* < 0.001, Fig. 2c). This successful CDC42EP3 knockdown in RKO and HCT116 cells was also verified by using western blotting (Fig. 2d). In all, these conclusions indicate that the lentivirus expressing shCDC42EP3 could significantly downregulate the expression of CDC42EP3 in colorectal cancer cells.

### CDC42EP3 development in vitro

**Fig. 3:**
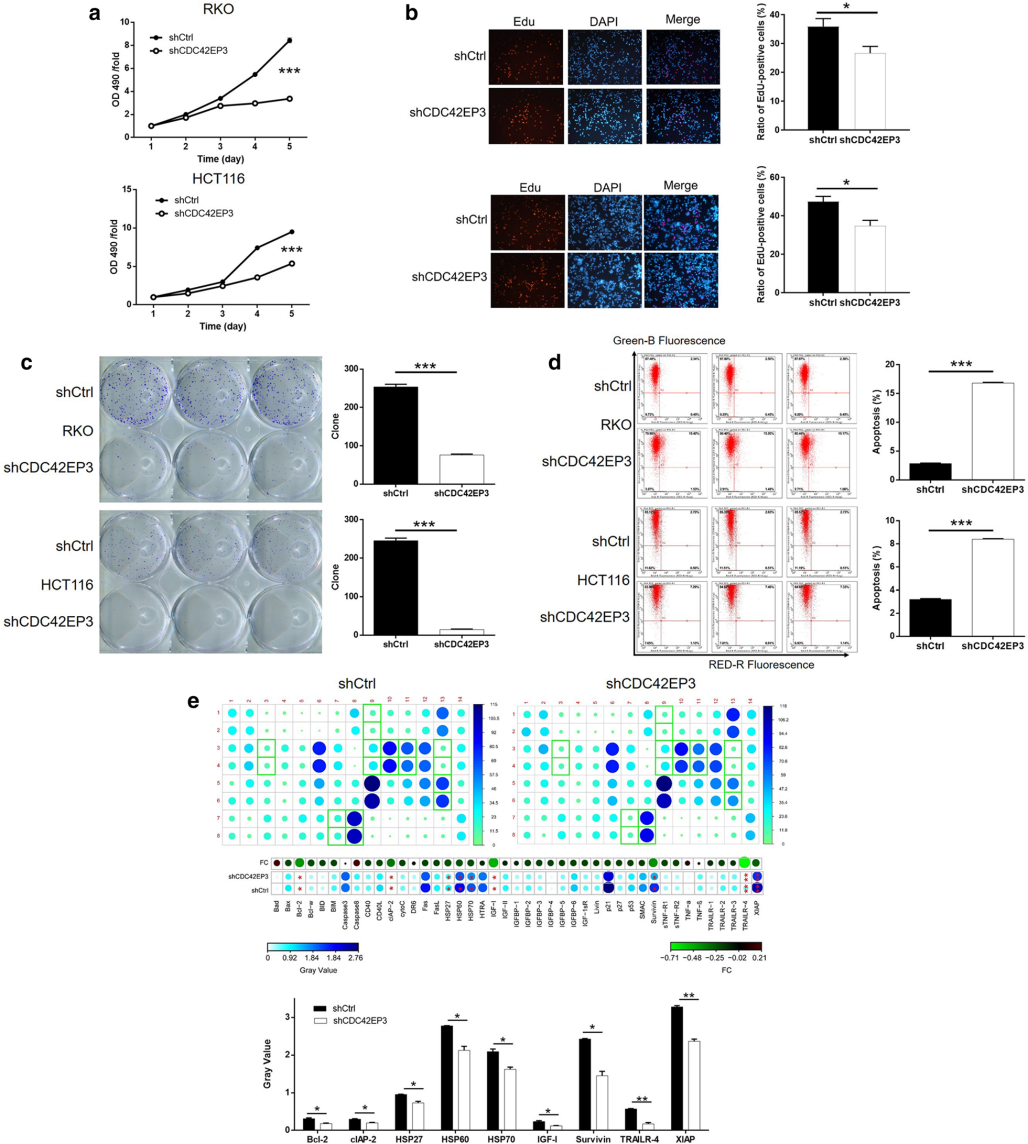
The effects of CDC42EP3 knockdown on cell proliferation, colony formation and cell apoptosis. **a** MTT was performed to estimate the effects of CDC42EP3 knockdown on cell proliferation. **b** EdU staining was performed to show the proliferative activity of RKO and HCT116 cells with or without CDC42EP3 knockdown. **c** Number of colonies formed by RKO and HCT116 cells with or without CDC42EP3 knockdown was counted. **d** Flow cytometry was employed to evaluate the cell apoptosis of RKO and HCT116 cells with or without CDC42EP3 knockdown. **e** Human Apoptosis Antibody Array was utilized to identify differentially expressed apoptosis-related proteins, by which CDC42EP3 regulated cell apoptosis. The representative images were randomly selected from at least 3 independent experiments in duplicate. *P < 0.05, **P < 0.01, ***P < 0.001

Detection in cellular function within RKO and HCT116 cells transfected with shCtrl or shCDC42EP3 revealed the contribution of CDC42EP3 to colorectal cancer development. Viability of colorectal cancer cells were demonstrated to be significantly lower in the shCDC42EP3 group during cell culture, indicative of the inhibition of cell proliferation (*P* < 0.001, Fig. 3a). Similar results were also observed through EdU staining assay. The ratio of EdU-positive cells in shCDC42EP3 was significantly reduced in comparison with shCtrl group (*P* < 0.05, Fig. 3b). The number of colonies, over an extended period of time, formed by cells transfected with shCDC42EP3 were notably less than that in shCtrl group (*P* < 0.001, Fig. 3c). Detection of cell apoptosis by flow cytometry revealed the significantly heightened apoptotic cell percentage in shCDC42EP3 group (*P* < 0.001, Fig. 3d). Additionally, through performing a Human Apoptosis Antibody Array we were able to explore how knockdown of CDC42EP3 affects cell apoptosis. The comparison between RKO cells with or without CDC42EP3 knockdown displayed a variety of significantly downregulated proteins, including Bcl-2, clAP-2, HSP27, HSP60, HSP70, IGF-I, Survivin, TRAILR-4 and XIAP (*P* < 0.05, Fig. 3e).

### CDC42EP3 knockdown suppressed cell motility through regulating EMT

**Fig. 4:**
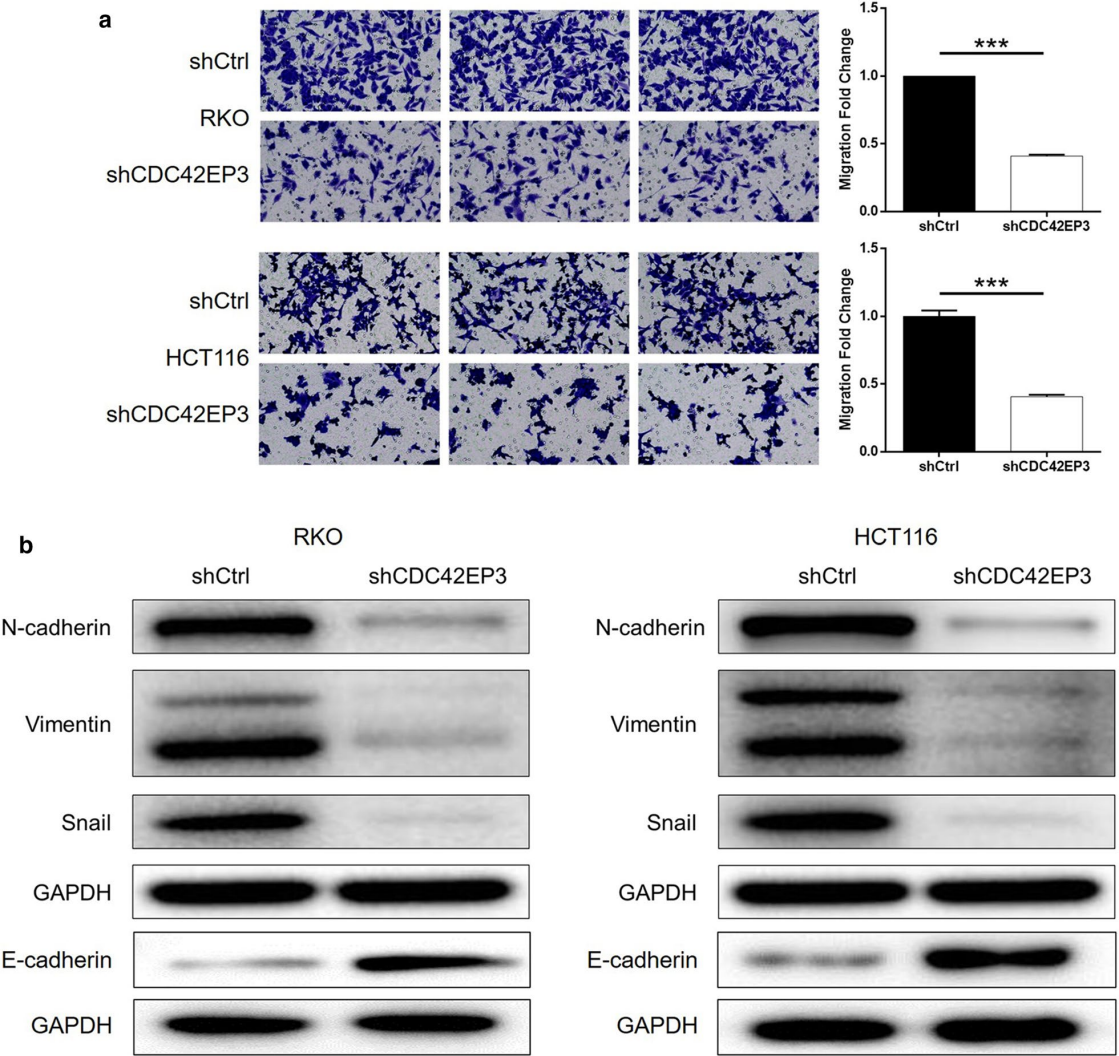
CDC42EP3 knockdown inhibited colorectal cancer cell migration through regulating EMT. **a** Transwell assay was conducted to study the influence of CDC42EP3 knockdown on cell migration of colorectal cancer cells. **b** The regulatory effects of CDC42EP3 knockdown on expression of EMT-related proteins were detected by western blotting. The representative images were randomly selected from at least 3 independent experiments in duplicate. *P < 0.05, **P < 0.01, ***P < 0.001

It is possible that CDC42EP3 depletion could regulate tumor metastasis of colorectal cancer, cell migration of RKO and HCT116 cells transfected with corresponding lentivirus was detected. Outcomes of Transwell assay reflected that knockdown of CDC42EP3 could significantly lower the cell migration capacity of RKO and HCT116 (*P* < 0.001, Fig. 4a). Additionally, easily recognized participations in tumor metastasis, epithelial-mesenchymal transition (EMT) related proteins including N-cadherin, E-cadherin, Vimentin and Snail were detected in RKO and HCT116 cells. Figure 4b highlights that the expression levels of N-cadherin, Vimentin and Snail showed substantial downregulation upon silencing of CDC42EP3, while E-cadherin was upregulated in shCDC42EP3 group, verifying the suppression of EMT as well as tumor metastasis by CDC42EP3 knockdown.

### In vivo verification of CDC42EP3 knockdown induced inhibition of colorectal cancer and mechanism exploration

**Fig. 5:**
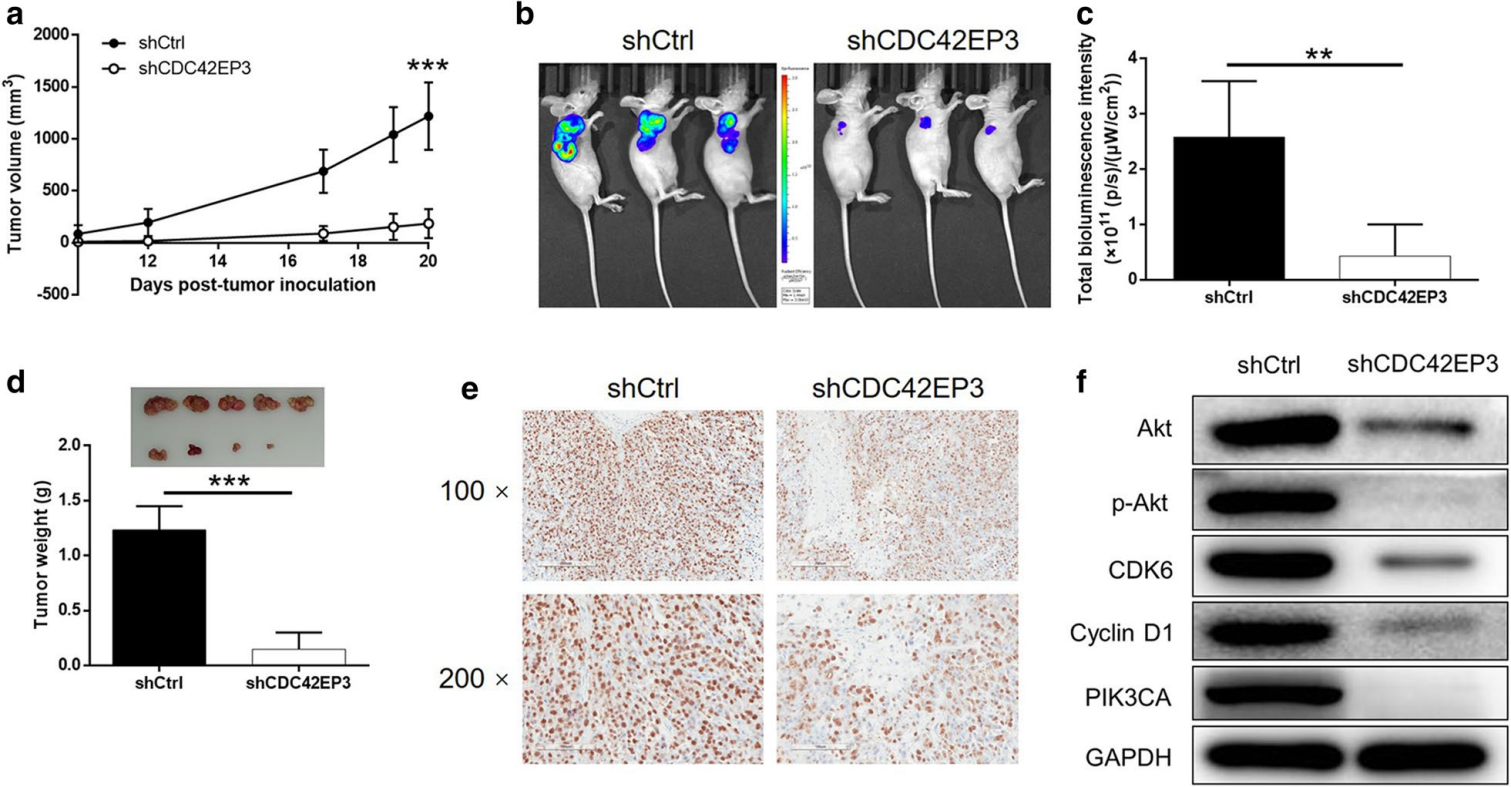
CDC42EP3 inhibited tumor growth of colorectal cancer in vivo. **a** The measurement of tumor volume started from day 10 post inoculation and ended at day 20 post inoculation. Mice were subjected to in vivo bioluminescent imaging (**b**), and the bioluminescence intensity was scanned and used to represent the tumor burden (**c**). **d** After sacrificing the mice, implanted tumors were removed, photographed, and weighted. **e** The expression of Ki-67 in tumors was detected by IHC. **f** The effects of CDC42EP3 knockdown on several cancer-related signaling pathways were estimated by western blotting. The representative images were randomly selected from at least 3 independent experiments in duplicate. *P < 0.05, **P < 0.01, ***P < 0.001

A mice-based xenograft model was constructed based on the subcutaneous injection of RKO cells with or without CDC42EP3 knockdown as a means to verify the role of CDC42EP3 in development of colorectal cancer in vivo. Measurements on the dimension of tumor size began at day 10 post inoculation and lasted for 10 days, the results indicated the decreased rate of growth of the tumors within the shCDC42EP3 group (Fig. 5a). The in vivo bioluminescence imaging facilitated by luciferase also displayed smaller and weaker tumors in shCDC42EP3 group (Fig. 5b, c). Upon removal of tumors from the mice, they were were photographed, weighted and subjected to IHC analysis of Ki-67. Figure 5d, e shows the tumors formed by RKO cells transfected with shCDC42EP3 were smaller, lighter and possessed a smaller volume of Ki-67. As such, it can be concluded that the inhibition of colorectal cancer by CDC42EP3 knockdown was proved in vivo. By contrast, the mechanism underlying the regulatory effects of CDC42EP3 on colorectal cancer were demonstrated through the detection of cancer-related signaling including Akt, CDK6, Cyclin D1 and PIK3CA, a reflection of the downregulation of Akt, p-Akt, CDK6, Cyclin D1 and PIK3CA (Fig. 5f).

## Discussion

Colorectal cancer is a malignant tumor with almost the highest morbidity and mortality worldwide [2, 17]. It is a malignant tumor originating from the colorectal mucosal epithelium [18]. So far, surgical resection is the only treatment that could radically cure colorectal cancer. However, it is limited by many factors such as tumor location, diagnosis time, disease classification, etc., and the cure rate is relatively low [19]. Recently, molecular targeted drug, which targets on the changes of tumor cell characteristics, have become one of the hottest topics in the field of cancer therapy. Molecular targeted drugs can not only exert stronger antitumor activity, but also reduce side effects on normal cells, which brings new hope for tumor treatment [20]. A variety of targeted drugs, such as bevacizumab (targeting VEGF), cetuximab and erlotinib (targeting EGFR), have been clinically applied in the treatment of colorectal cancer [21, 22]. Nevertheless, the prognostic improvement of colorectal cancer patients is still far from satisfactory. Therefore, continuous exploring novel therapeutic targets of colorectal cancer could not only benefit for the understanding the underlying molecular mechanism of colorectal cancer progression, but also facilitate the development of targeted drugs with better specificity and anti-cancer efficiency [23–25].

Rho protein family is an important hub of signal transduction in cells, which can quickly convert between the activation state of GTP binding and the inactivation state of GDP binding, transmit the extracellular signal to the cells, and act as a ‘molecular signal switch’ [26]. So far, more than 10 proteins have been found in three subfamilies of Rho protein family, including Cdc42 [27, 28]. In addition to its function in normal cell activities, the abnormal high expression of Cdc42 in a variety of malignant tumors has been closely related to the occurrence, development, invasion and metastasis of malignant tumors [9]. For instance, Razidlo et al*.* delineated that Cdc42 could be rapidly and robustly activated by a pro-inflammatory factor Interleukin-6 (IL-6), thus mediating the IL-6 induced promotion of tumor growth and metastasis [29]. Cdc42 and its relevant signaling pathways were also widely studied as target of small molecules and microRNAs in cancer [30]. CDC42EP3 is a member of the BORG/CDC42EP family of Cdc42 effectors, which includes 5 members [31, 32]. Till now, the studies concerning the function of CDC42EP3 mainly aimed on its role in cancer-associated fibroblasts (CAFs). Research of Fernando’s group revealed the essential role of CDC42EP3 in matrix remodeling, invasion, angiogenesis and ability to promote tumor growth of CAFs, which was tightly regulated by Cdc42 [13, 15]. However, except for this, the association between CDC42EP3 and human cancer, including colorectal cancer is still largely unknown, the exploration of which could extend the understanding of CDC42EP3 in cells.

The current study aimed to exam the potential link between CDC42EP3 and colorectal cancer. First, we detected CDC42EP3 expression in colorectal cancer tissues relative to normal tissues and found significantly upregulated CDC42EP3 in former by IHC analysis. Further analysis revealed that high CDC42EP3 expression was positively linked to advanced tumor grade, indicating CDC42EP3 as a tumor promotor in colorectal cancer. The subsequent in vitro experiments indicated that CDC42EP3 knockdown in colorectal cancer cells significantly slowed down the growth rate, increased the percentage of apoptotic cells via the activation of apoptosis-related proteins, and hindered cell migration. At the same time, the data from in vivo experiments based on mice xenograft model were in line with those from in vitro experiments, consistently reflecting the suppression effects of CDC42EP3 knockdown on the development and progression of colorectal cancer.

Tumor invasion and metastasis are inseparable from epithelial-mesenchymal transition (EMT) [33], which means that tumor cells change from epithelial phenotype to mesenchymal phenotype, and move from primary focus to metastatic pathway, thereby completing tumor invasion and metastasis [34]. Studies have shown that the occurrence of EMT simultaneously followed by the downregulation of E-cadherin and upregulation of N-cadherin, which might accelerate tumor invasion and metastasis [35]. Consistently, our study provided an evidence that the inhibition of cell migration caused by CDC42EP3 deficiency could be restored to a reasonable level by regulating the expression of N-cadherin and E-cadherin. On the other hand, Vimentin also played an important role in mesenchymal cells and epithelial cells. It was presented by Zhu et al*.* that CircNHSL1/miR-1306-3p/SIX1 axis could affect the development and progression of gastric cancer by cooperating with Vimentin and EMT [36]. Similarly, we found that CDC42EP3 depletion in cells resulted in the down-regulation of Vimentin expression. Otherwise, Snail is an identified transcription factor that is upregulated in EMT, and in our study it was shown that Snail expression was suppressed by CDC42EP3. Given the above all results, it was suggested that CDC42EP3 had the ability to regulate EMT in colorectal cancer.

Akt-mediated signaling is known for its involvement in the biological behavior of tumor cells, such as proliferation, differentiation, apoptosis and migration [37–39]. It followed that in our study the inhibition of CDC42EP3 obviously down-regulated the expression of Akt and phosphorylated Akt. Additionally, it was reported that both Cyclin D1 and CDK6 were important regulator in cell cycle and cell proliferation [40, 41]. The prior studies have revealed that Cyclin D1 and CDK6 were abundantly expressed in colorectal cancer and have attracted much attention in multiple areas of cancer research and prognosis judgement [42, 43]. Identical results were obtained in our study in which both Cyclin D1 and CDK6 showed significantly downregulation upon knockdown of CDC42EP3. Moreover, PIK3CA, a well-known cancer-related protein [44], was also found to be downregulated by CDC42EP3 knockdown.

## Conclusions

In conclusion, our study revealed that CDC42EP3 played a critical role in the development and progression of colorectal cancer. Knockdown of CDC42EP3 could effectively inhibit cell proliferation through inducing cell apoptosis. Therefore, CDC42EP3 may be used as novel therapeutic target in the treatment of colorectal cancer.

## Data Availability

Not applicable.
